# Novel C3 mutation p.Lys65Gln in aHUS affects complement factor H binding

**DOI:** 10.1007/s00467-012-2183-z

**Published:** 2012-06-06

**Authors:** Elena Volokhina, Dineke Westra, Xiaoguang Xue, Piet Gros, Nicole van de Kar, Lambert van den Heuvel

**Affiliations:** 1 Department of Pediatric Nephrology (804), Radboud University Nijmegen Medical Centre, P.O. Box 9101, 6500 HB Nijmegen, The Netherlands; 2 Department of Crystal and Structural Chemistry, Bijvoet Center for Biomolecular Research, Utrecht University, Utrecht, the Netherlands; 3 Department of Laboratory Medicine, Radboud University Nijmegen Medical Centre, Nijmegen, the Netherlands; 4 Department of Pediatrics, University Hospitals Leuven, Leuven, Belgium

**Keywords:** aHUS, C3, Complement regulation, DNA analysis, Genetic defects

## Abstract

**Background:**

Atypical hemolytic uremic syndrome (aHUS) is associated with mutations affecting complement proteins and regulators and with autoantibodies against complement factor H (CFH). Approximately half of the aHUS patients progress to end-stage renal disease. DNA analysis of the risk factor genes is important for prognosis of aHUS recurrence after renal transplantation.

**Methods:**

Mutational screening of *C3* encoding the central complement component was performed by Sanger sequencing in 70 aHUS patients. Mutated and wild type recombinant C3b proteins were produced and their affinity to CFH was analyzed by ELISA.

**Results:**

A single novel missense change p.Lys65Gln in C3 was found in 3 aHUS patients. The alteration leads to decreased binding of C3b to CFH in vitro. All three patients acquired the illness as adults and had a first aHUS episode after renal transplantation or suffered recurrence of the disease after transplantation.

**Conclusions:**

The novel *C3* change was found in 3 aHUS patients. It results in decreased C3b binding to CFH and thus might lead to impaired C3b inactivation in vivo. The p.Lys65Gln is likely to be associated with aHUS after kidney transplantation and, therefore, might be an important prognostic factor.

## Introduction

Hemolytic uremic syndrome (HUS) is characterized by hemolytic anemia, thrombocytopenia, and acute renal failure [1]. In most cases, HUS is preceded by infection with Shiga-like toxin-producing *Escherichia coli* (*STEC*). Five to 10% of all HUS patients acquire the disease without being infected with *STEC*. These atypical HUS (aHUS) patients have a poor prognosis, with up to 50% of cases progressing to end-stage renal disease (ESRD) and up to 25% of lethal outcomes in the acute phase [2]. Furthermore, HUS can occur with a variety of causes, including non-enteric infections (*Streptococcus pneumoniae*), use of medication, and pregnancy [3–5].

The aHUS etiology has been linked to ongoing alternative complement pathway activation. In this alternative pathway, complement component C3 is spontaneously activated at a very low rate to form C3b. The C3b is able to attach to the surfaces of pathogens and host cells, where it binds complement factor B (CFB), which in turn is cleaved by complement factor D (CFD). The resulting C3bBb or C3 convertase cleaves and activates C3 leading to amplification of the complement cascade, to the formation of a membrane attack complex, and, eventually, to cell lysis. At the surface of the normal host cells C3b is cleaved by complement factor I (CFI), while complement factor H (CFH), membrane cofactor protein (CD46/MCP), and complement receptor type 1 act as cofactors. In addition, at the surface of the normal host cells C3 convertase can be dissociated by regulators [6]. Mutations affecting CFH, CFI, MCP, C3, CFB, thrombomodulin and the presence of autoantibodies against CFH [7–14] are associated with aHUS pathogenesis. Complement deficiencies are identified in 50–60 % of aHUS patients [15, 16]. In particular, new C3 mutations, affecting C3 convertase in a gain-of-function manner, were recently described [17, 18].

Etiological analysis of patients with aHUS is very important, especially in renal transplantation, which is frequently required in this patient group. For example, patients that carry mutations in genes encoding CFH or CFI are at higher risk of the disease recurrence in the graft (70–90%), whereas such probability is much lower for the aHUS patients carrying MCP mutations (20%) [15, 16]. Previously, we reported prevalence of mutations in CFH, CFI, MCP, and CFB, and autoantibodies against CFH in Dutch/Belgian aHUS cohort [19]. In this study we report C3 variations found in our aHUS patients.

## Materials and methods

### Study population

The research population consisted of 70 aHUS patients (age 2 months to 52 years at onset of the disease), referred to the Pediatric Nephrology Department of the Radboud University Nijmegen Medical Centre. All patients were of Dutch or Belgian origin and diagnosed with non-*STEC*-HUS. In 15 patients from 10 families the familial form of aHUS was identified; the other 55 patients were diagnosed with sporadic aHUS. Informed consent of all patients or their parents was obtained before the DNA analysis. The missense *C3* alterations found in patients were also analyzed in genomic DNA from 100 healthy, ethnically-matched control individuals.

### Sequence analysis of the C3 gene

Genomic DNA was isolated from peripheral blood leukocytes as described by Miller et al. [20]. Fragments of the *C3* gene (NCBI mRNA RefSeq NM_000064.2, genomic RefSeq NG_009557.1 [21]) were amplified from genomic DNA by means of PCR. Primer sequences are available upon request. The PCR products obtained included DNA sequences of the 41 individual exons, flanked by the splice donor site and the splice acceptor site. The amplimers were subjected to double-stranded DNA sequence analysis on an ABI 3130 *xl* GeneticAnalyzer (Applied Biosystems). Sequence analyses were performed using Sequencher 4.8 software (Gene Codes). Sorting Intolerant From Tolerant (SIFT) (http://sift.jcvi.org/) [22] and PolyPhen-2 (http://genetics.bwh.harvard.edu/pph2/) [23] mutation analysis algorithms were used to access potential pathogenicity of C3 changes on protein level. The SIFT scores were obtained by submitting C3 protein sequences of *Homo sapiens*, *Bos taurus*, *Sus scrofa*, *Canis familiaris*, *Rattus norvegicus*, *Mus musculus*, and *Xenopus tropicalis* to the program. Substitutions with scores below the threshold of 0.05 are considered intolerant by SIFT and are likely to affect protein function.

### Recombinant C3b production

DNA fragment encoding mature wild-type C3 protein was cloned into the PCR4-TOPO (Invitrogen), the c.193A>C (p.Lys65Gln) and c.481C>T (p.Arg161Trp) sequence variations were introduced using the QuikChange method. Subsequently, the C3 variants were sub-cloned into a modified pUPE expression vector (U-Protein Express BV). The wild-type and mutant C3 constructs were expressed in HEK293-E cells in the presence of furin to ensure correct C3 processing [24]. After 3 days of expression medium was collected and centrifuged (1000 *g*, 15 min, 4°C), supernatant was used in experiments. The expression levels of C3 in the medium were 2–7 μg/mL. C3b was generated from C3 using CFB and CFD. The medium samples containing 1 μg/mL C3 were incubated with 1.8 μg/mL CFB and 0.13 μg/mL CFD (both from Complement Technology) for 2 h at 37°C. Cleavage of C3a was verified by SDS-PAGE.

### Binding affinity assay

The binding affinity assay was done in an ELISA setting. Wells of the ELISA plates were coated with 1 μg/mL of purified CFH (Calbiochem). The coated wells were incubated with medium samples containing 1,000, 500, 250, or 125 ng/mL of wild-type or mutant C3b and the presence of C3b was detected using horseradish peroxidase (HRP)-conjugated goat antibodies against C3 (MP Biomedicals).

### Statistical analyses

The statistical significance of allelic frequencies was analyzed using two-tailed Fisher's exact test. The statistical analysis of the binding affinity assay results was performed using two-way ANOVA. The differences with *P* < 0.05 were considered statistically significant.

## Results

### *C3* DNA alteration identified in three aHUS patients

**Fig. 1:**
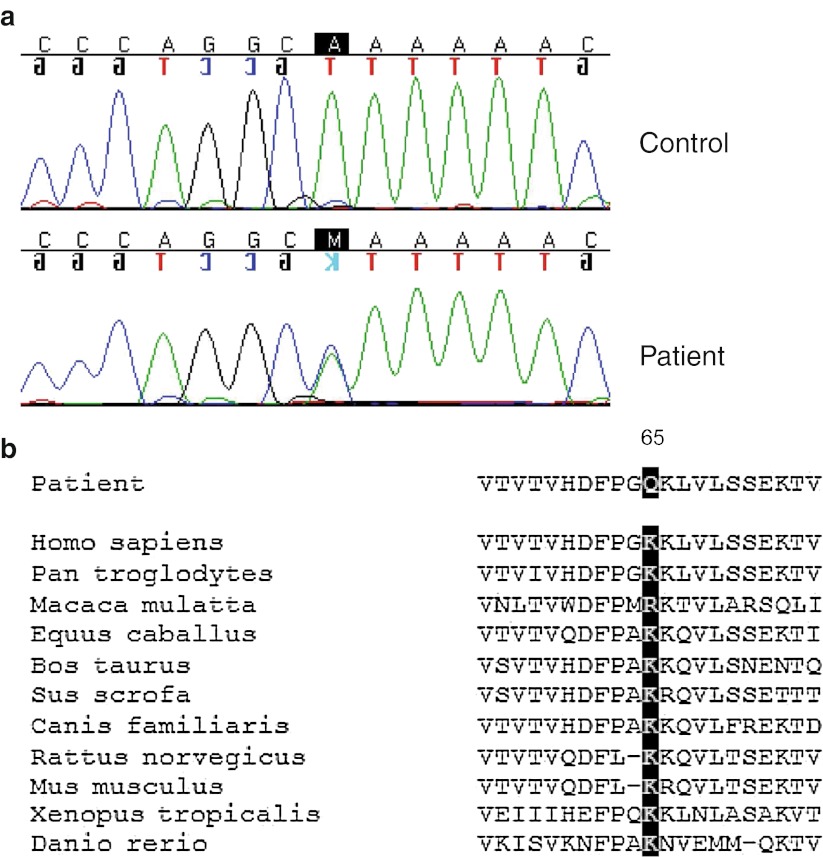
Novel sequence variation found in 3 atypical hemolytic uremic syndrome (aHUS) patients. **a** Sequencing results of a healthy control (*upper panel*) and a patient (*lower panel*). Location of c.193A>C is indicated by a *black box*. **b** Sequence alignment of the C3 protein regions from various species containing Lys65, which is altered in aHUS patients. Altered amino acid position is marked by a *black box* and its number is indicated

The total open reading frame of the *C3* gene was analyzed in 70 patients with aHUS. A novel missense DNA change c.193A>C, leading to p.Lys65Gln substitution was identified in 3 patients in heterozygous form (Fig. 1a, Table 1). All of the patients had a sporadic form of the disease and did not carry mutations affecting CFH, CFI, MCP, CFB, thrombomodulin or autoantibodies against CFH. The novel change was not found by us among 100 healthy controls, nor is it reported in dbSNP. It is also not reported in the NHLBI Exome Sequencing Project (ESP), which carries whole exome sequencing data from over 5000 human exomes (http://evs.gs.washington.edu/EVS/). The p.Lys65Gln alteration affects a conserved residue (Fig. 1b). It has a SIFT score of 0.00 and is predicted to be probably damaging by PolyPhen-2, indicating intolerance and a possible impact on the C3 structure and/or function. We also encountered a recently described p.Arg161Trp [17] (referred to by the authors as p.Arg139Trp, while not counting the signal peptide) in 11 patients in heterozygous form. Interestingly, although this change was not found by us among 100 controls, we did detect it in 3 healthy parents of aHUS patients, while the patients themselves did not possess the change. The prevalence of this polymorphism among aHUS patients was significantly higher (*p* = 0.01) than among the healthy individuals screened.

**Table 1:** Clinical data available for patients carrying p.Lys65Gln

Patient number	Gender (F/M)	Age at onset	C3 levels in acute aHUS phase^a^	Transplantation history	Outcome
1	M	40	0.73–0.95	aHUS after transplantation^b^	Partial recovery
2	F	18	0.48–0.76	aHUS after transplantation^c^ and aHUS recurrence in second graft	ESRD
3	M	45	0.5	Transplantation after aHUS and aHUS recurrence in the graft	ESRD

*aHUS* atypical hemolytic uremic syndrome, *ESRD* end-stage renal disease

^a^C3 normal values: 0.70–1.50 g/L

^b^Kidney transplantation related to thrombotic microangiopathy as a result of malignant hypertension

^c^Kidney transplantation related to rapidly progressing glomerulonephritis

### p.Lys65Gln decreases C3b binding to CFH in vitro

**Fig. 2:**
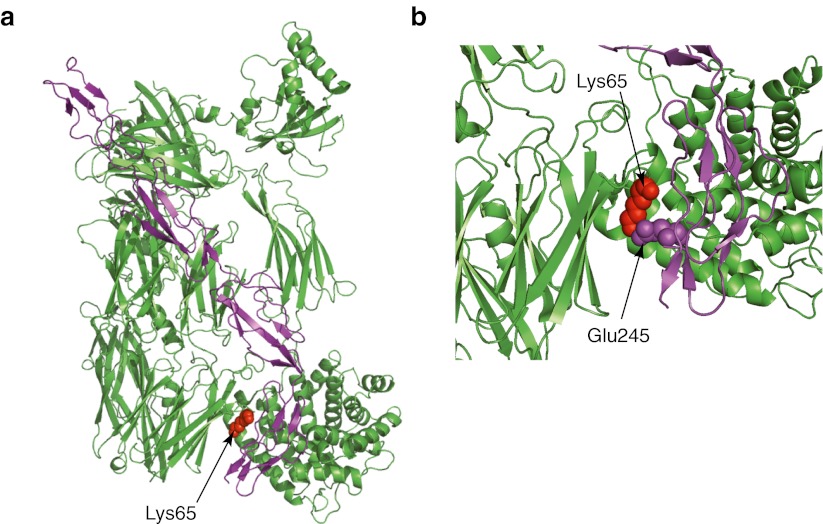
Location of Lys65 amino acid altered by the missense mutation in the atypical hemolytic uremic syndrome (aHUS) patients. C3b domains are colored in green and complement factor H (CFH) domains are shown in purple. Amino acid residue mutated in aHUS patients is indicated by red spheres. **a** Structure of C3b in complex with CFH domains 1–4 [25]. **b** Enlarged image showing direct interaction of Lys65 residue of C3b with Glu245 residue of the CFH. The images were generated using PyMol

**Fig. 3:**
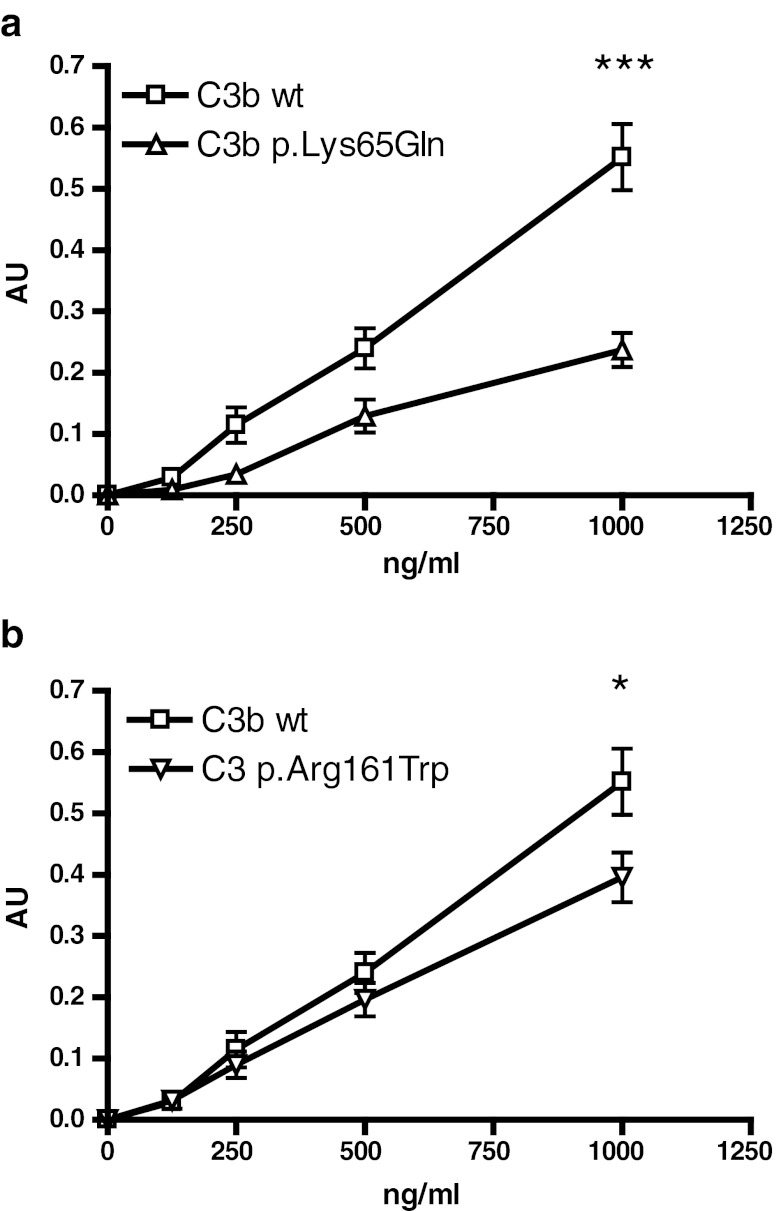
C3b binding affinity to complement factor H (CFH). ELISA plates were coated with purified CFH, after that the wells were incubated with various concentrations of the recombinantly produced wild-type and **a** p.Lys65Gln or **b** p.Arg161Trp C3b variants. Binding of the C3b variants was detected using antibodies against C3. ELISA results are expressed in arbitrary units (AU). The data represent four independent experiments and are presented as mean ± SE. Significant differences according to ANOVA with *p* < 0.001 (***) and *p* < 0.05 (*) are indicated

The altered amino acid is located in the part of the *C3* gene encoding C3b, at the interface of the C3b and CFH domain 4 (Fig. 2a) [25]. Lysine, at position 65 in C3b, is in direct contact with the CFH glutamate at position 245 (Fig. 2b). These C3b and CFH residues are forming a salt bridge, a relatively weak ionic bond between positively charged lysine and negatively charged glutamic acid. Replacement of a lysine with a glutamine might, therefore, weaken the interaction between CFH and C3b. To test this hypothesis, recombinant C3 protein, carrying p.Lys65Gln, was produced and cleaved using CFB and CFD to yield C3b. Binding of the recombinant mutant and wild-type C3b to purified CFH was compared in an ELISA setting (Fig. 3a). The p.Lys65Gln change resulted in a statistically significant (*p* < 0.001) decrease in CFH binding when C3b concentration reached 1,000 ng/mL. This finding indicates that the DNA alteration leads to the weaker affinity of C3b to CFH.

In contrast to the previously reported data [17], in a similar experiment we also observed a weaker binding for C3b variant carrying p.Arg161Trp (Fig. 3b). Although this decrease is less pronounced than that of p.Lys65Gln, it is statistically significant (*p* < 0.05).

### p.Lys65Gln is found in adult patients with aHUS in a kidney graft

All patients carrying p.Lys65Gln were adults at the time of the first aHUS episode.

The first patient was initially diagnosed with thrombotic microangiopathy as a result of malignant hypertension in 2003. Hemolytic anemia and thrombocytopenia were not found, the thrombotic microangiopathy (TMA) diagnosis was made based on the biopsy results. The patient was referred for hemodialysis and later received a living-donor kidney from his sister. Five months later, the patient developed thrombotic microangiopathy combined with declining renal function. Serum C3 levels were at the lower border of the normal range (Table 1) and the patient was diagnosed with aHUS. Incomplete recovery of the kidney function after this episode was achieved.

The second patient initially suffered from rapidly progressing glomerulonephritis (RPGN) of undefined etiology in 2003. The laboratory findings show no hemolytic anemia or thrombocytopenia, renal biopsy was not performed; therefore, the presence of TMA was not determined. The patient received a living-donor kidney transplant from her father. She developed aHUS in this renal graft and also in the next cadaver kidney transplant. In the third cadaver kidney transplant the patient developed acute tubular necrosis and was referred for hemodialysis. Eventually, the patient died of Gram-negative septic shock.

The third patient developed TMA, diagnosed by renal biopsy, in combination with low C3 levels in serum. The patient received a kidney transplant, but 6 months later aHUS recurred in the graft.

Interestingly, in all 3 patients aHUS (re)occurred after kidney transplantation.

## Discussion

In this study, a novel missense sequence variation c.193A>C was found leading to p.Lys65Gln substitution in the *C3* gene. The mutation alters a highly conserved amino acid (Fig. 1b). All of the analyzed species carry a lysine at position 65, except for *Macaca mulatta*, where its place is taken by an arginine, which is, similar to lysine, a positively charged hydrophilic amino acid. On the contrary, a glutamine, found at this position in aHUS patients has a neutrally charged side chain. As shown by our data, the replacement of lysine with glutamine compromises C3b-CFH interaction. It might lead to the decreased rate of C3b cleavage by CFI and decreased dissociation of C3 convertase by CFH decay-acceleration activity in vivo. Inefficient complement inactivation at the cell surface would result in damage of the endothelium of the glomeruli.

Further experiments should be considered for future analysis to increase the impact of the functional role of mutation, such as measurement of complement activation products in the serum of controls and patients carrying the mutation. Furthermore, complement deposition on human glomerular microvascular endothelial cells and human umbilical vein endothelial cells from patient and control serum can be compared.

Clinical data were available for all 3 patients. All of the patients acquired aHUS in renal transplants. This finding is important, because it indicates that the p.Lys65Gln substitution in C3 might be associated with poor prognosis in renal transplantation.

The previously described aberration p.Arg161Trp was found in 11 patients and in 3 healthy parents of other aHUS patients who did not possess the change themselves. The incidence of the p.Arg161Trp substitution is significantly higher in the aHUS group than among the healthy individuals. This indicates that p.Arg161Trp is rather an aHUS-predisposing single nucleotide polymorphism than an aHUS-causing mutation. Furthermore, our data indicate a significant weakening of the C3b-CFH interaction by p.Arg161Trp. This weakening was not observed previously [17]. Roumenina et al. used a concentration range 0–300 ng/mL of C3b, while in our studies we used a broader C3b concentration range and observed a significant weakening of CFH binding at 1,000 ng/mL C3b [17]. Our findings provide new insight into the pathogenicity mechanism of p.Arg161Trp, a strongly predisposing aHUS polymorphism.

Previously, we reported genetic aberrations found in *CFH*, *CFI*, *CFB*, and *MCP*, and the presence of autoantibodies against CFH in our aHUS patient cohort [19]. In this study, we described a potentially pathogenic p.Lys65Gln mutation in the *C3* gene in our patients. Moreover, in 11 patients we identified a p.Arg161Trp polymorphism, which strongly predisposes to aHUS. In total, the prevalence of C3 changes in the Dutch/Belgian aHUS cohort is 20% (14 out of 70). Together with the previously reported findings [19], 48.6% (34 out of 70) of patients in our aHUS cohort display potential disease-causing alterations in genes encoding complement (regulating) proteins.
